# Parasitoid Abundance and Community Composition in Desert Vineyards and Their Adjacent Natural Habitats

**DOI:** 10.3390/insects11090580

**Published:** 2020-09-01

**Authors:** Michal Segoli, Miriam Kishinevsky, Tamir Rozenberg, Ishai Hoffmann

**Affiliations:** 1 Mitrani Department of Desert Ecology, The Jacob Blaustein Institutes for Desert Research, Ben-Gurion University of the Negev, Sede Boqer Campus 8499000, Israel; tamirro@bgu.ac.il; 2 Department of Evolutionary and Environmental Biology, University of Haifa, Haifa 3498838, Israel; mashakish@gmail.com (M.K.); ishaih@bgu.ac.il (I.H.)

**Keywords:** parasitoid, conservation biological control, vineyard, community composition, desert habitat

## Abstract

**Simple Summary:**

Desert agricultural systems are characterized by extreme contrast in environmental conditions between the irrigated fields and their surrounding natural habitats. We compared insect communities between vineyards and their surrounding desert habitats, in a hyper-arid region in Israel. We focused on parasitoid wasps—a highly diverse group with members that are important for the biological control of insect pests. Parasitoids were more abundant outside of the vineyard at the beginning of the vine growth season and became more abundant within the vineyard habitats later in the season. In contrast to our predictions, many parasitoid species were found both within and outside of the vineyards. This highlights the importance of the natural surrounding habitats in maintaining and providing resources for potentially beneficial biological control agents and calls for their preservation.

**Abstract:**

Parasitoids are important natural enemies of many agricultural pests. Preserving natural habitats around agricultural fields may support parasitoid populations. However, the success of such an approach depends on the ability of parasitoids to utilize both crop and natural habitats. While these aspects have been studied extensively in temperate regions, very little is known about parasitoid communities in desert agroecosystems. We took one step in this direction by sampling parasitoids in six vineyards and their surrounding natural desert habitat in a hyper-arid region of the Negev Desert Highlands, Israel. We predicted that due to the high contrast in environmental conditions, parasitoid abundance and community composition would differ greatly between the crop and the natural desert habitats. We found that parasitoid abundance differed between the habitats; however, the exact distribution pattern depended on the time of year—with higher numbers of parasitoids in the natural habitat at the beginning of the vine growth season and higher numbers in the vineyard at the middle and end of the season. Although parasitoid community composition significantly differed between the vineyard and desert habitats, this only accounted for ~4% of the total variation. Overall, our results do not strongly support the notion of distinct parasitoid communities in the crop vs. the desert environment, suggesting that despite environmental contrasts, parasitoids may move between and utilize resources in both habitats.

## 1. Introduction

Conservation biological control relies on the preservation of natural enemies (e.g., predators and parasitoids) of agricultural pests, in order to reduce pest populations and, consequently, crop damage [1]. One way this can be achieved is by preserving natural or semi-natural habitats in proximity to crop fields [2,3,4]. Non-crop habitats may potentially provide natural enemies with resources, such as nectar, shelter, and alternative prey, thereby increasing their abundance and efficiency in controlling pest populations [5]. However, for this approach to be successful, natural enemies should be able to exploit resources both within and outside the crop fields. This ability may depend on their mobility, but also on the environmental contrast between these habitat types [6].

While the importance of preserving natural habitats for conservation biological control has been studied extensively in temperate agroecosystems, very little is known about the effectiveness of this approach in arid regions e.g., see map in [7]. Yet, most of our planet consists of arid and semi-arid regions, which are likely to expand even further due to climate change and desertification processes [8]. Desert agroecosystems may differ from temperate ones in the degree of environmental contrast between the irrigated, fertilized crop areas and the relatively dry and barren natural habitats. Such large contrasts may restrict the movement of natural enemies between the crop and non-crop habitats, due to their adaptations to specific environmental conditions [5,6,9]. This may result in distinct community compositions between the two contrasting habitats and, potentially, limit the advantage of preserving natural habitats for conservation biological control. 

Parasitoid wasps are among the most diverse groups of animals [10] and play an important ecological role in controlling insect populations [11]. Moreover, parasitoids are considered primary natural enemies of many agricultural pests [12]. Previous studies of parasitoid communities in agroecosystems provide evidence that increased habitat diversification, and, in particular, the preservation of non-crop habitats around agricultural fields can increase parasitoid abundance and diversity, as well as parasitism rates of key agricultural pests [13,14,15,16,17,18,19]. However, the influence of habitat diversification on parasitoid communities in desert agroecosystems has rarely been investigated [20].

To address this knowledge gap, we studied parasitoid communities in and near vineyards in the hyper-arid region of the Negev Desert Highlands in Israel. In historical times, this area was occupied by agricultural settlements located along the ancient Incense Trade Route. These settlements maintained agricultural orchards and vineyards by diverting and preserving rainwater [21,22]. More recently, this area was settled by several private farms that grow mainly grapes and olives using irrigation water. The farms are largely surrounded by natural or semi-natural desert areas. However, the importance of these adjacent habitats in supporting potential natural enemies of vineyard pests has not been examined previously. 

To this end, we sampled parasitoids in six vineyards and their adjacent natural habitats, from both naturally occurring vegetation and grape leaves. We distinguished between two types of desert habitats: the riverbed (wadi), which accumulates water in the wet season; and the wadi slope, which is generally drier [23]. In addition, we distinguished between two types of vineyards habitats: the center of the vineyard; and the vineyard edge, which may represent a distinct habitat [2,24]. We hypothesized that due to the sharp contrast in resource availability and other environmental variables, parasitoid assemblages will differ greatly between the agricultural habitats and the natural habitats. More specifically, we predicted that parasitoid abundance will be higher in the agricultural habitats than in the desert habitats, especially later in the season (summer months), when the natural vegetation mostly dries out. We also predicted that parasitoid species composition will differ significantly between the habitat types, reflecting specific adaptations and habitat preference by different species. We consider this study as a further step towards characterizing parasitoid communities in desert agroecosystems, most likely the first that focuses on vineyards in a hyper-arid region. 

## 2. Methods

### 2.1. Study Area and Sampling Design

Parasitoids were sampled in six vineyards in the Mitzpe Ramon area of Israel’s Negev Desert Highlands (~700-m elevation). This area is characterized by a hyper-arid climate with ~80 mm of winter rains yearly, hot and dry summers with 18–30 °C (mean min-max) daily temperatures, and cold winters with 7–13 °C daily temperatures (the Israel Meteorological Service; http://www.ims.gov.il/IMSEng/CLIMATE). The grape-growing season in this region starts in March–April (start of leaf growth) and lasts till August–September (harvest).

**Figure 1:**
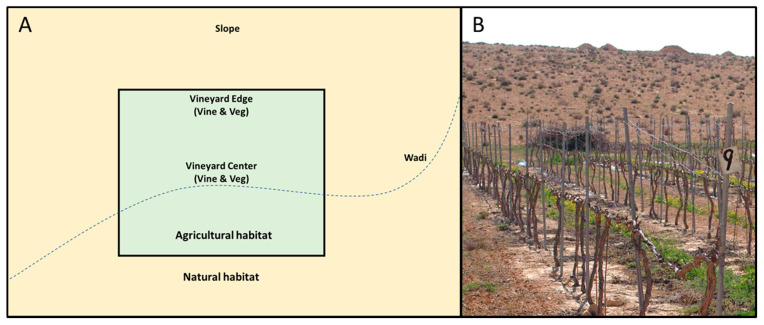
(**A**) Schematic illustration of the experimental design. In each vineyard, two agricultural habitats were sampled: the vineyard center, and the vineyard edge, where both ground vegetation (veg) and grape foliage (vine) were suction-sampled. In addition, two natural habitats were sampled: the wadi riverbed and the slope, where natural vegetation was suction-sampled. (**B**) Photograph of one of the study vineyards and the surrounding natural habitat (slope) in March 2015.

Sampled vineyards were surrounded by natural or semi-natural desert habitats (Figure 1), but differed in size, age, and management practices as summarized in Table S1. Parasitoids were suction-sampled from the vegetation using a Vortis Insect Suction Sampler (Burkard Manufacturing Co. Ltd., Rickmansworth, UK). The starting point for each sampling was chosen randomly, and insects were sampled from all the green vegetation encountered during continuous 30 s of gradual walking within the relevant habitat (covering an approximate area of ~3 m^2^) while avoiding resampling the same section or plant. For each sample, we also recorded the identity of plant species that were present in the sampled section. This was done by an expert directly in the field, or in cases of uncertainty, a plant was taken to the lab for further identification (see Table S2 for a full list of plant species). In each sampling event, two points were sampled in each of the following locations (see Figure 1): from the ground vegetation in the vineyard center (*veg-center*); from the grape foliage in the vineyard center (*vine-center*); from the ground vegetation in the vineyard edge (*veg-edge*); from the grape foliage in the vineyard edge (*vine-edge*); from the natural vegetation in a nearby slope (~50–100 m from the vineyard edge) (*slope*); and from the natural vegetation in a nearby dry streambed (~50–100 m from the vineyard’s edge) (*wadi*). Samples of these different locations were taken in March, June, and August 2015, with the exception of grape foliage samples that were not available in March (prior to grape leaf emergence). Altogether, 192 samples were taken (see Table 1 for details). Samples were stored in 75% ethanol and refrigerated until sorting. Parasitoid wasps were classified to genus or species using various keys [25,26,27,28,29,30,31,32,33,34,35,36,37].

### 2.2. Statistical Analyses

Since two samples were taken in each habitat and date, we considered them as pseudo-replications and first averaged the number of parasitoids in each pair for the purpose of the abundance analyses. To test the effects of the month (March/June/August), habitat (center/edge/slope/wadi), type of vegetation (veg/vine foliage), and the number of plant species in each sampling location on general parasitoid abundances, we used zero-inflated generalized linear mixed odels (GLMM) with a negative binomial distribution and a log-link function. This type of model was chosen because many of the samples contained no parasitoids, and the data distribution deviated from Poisson. The total parasitoid abundance in each averaged sample was used as a dependent variable with month, habitat, vine vs. vegetation, and plant species richness (the number of plant species that were recorded in the sampled area) as fixed effects, and the site as a random-intercept factor. Likelihood ratio tests were used to compare the model with and without each explanatory variable. The variables were excluded one at a time and the resulting reduced models were compared to the full model. Additional models were used to test the effects of the same explanatory variables on the abundance of the dominant parasitoid species. We were able to run these tests only for the five most abundant species, where sample sizes were large enough to avoid model overfitting. To test the effects of the same independent variables as used in the GLMM on the community composition, we used a permutational multivariate analysis of variance (Adonis test), which takes into account both species presence and abundance [38,39], with site as a stratifying variable. The packages “vegan” [40,41] and “glmmTMB” [42] were used; analyses were carried out in R version 3.4.0 (R Core Team 2013, http://www.R-project.org/).

## 3. Results

### 3.1. Parasitoid Families

Overall, 1570 individual parasitoid wasps were sampled, representing 155 morpho-species of 17 families. Trichogrammatidae was the most common family (37%) followed by Mymaridae (13%), Platygastridae (13%), Eulophidae (11%), Pteromalidae (8%) Encyrtidae (7%), and Braconidae (4%), while all other families combined accounted for less than 10%.

### 3.2. Parasitoid Abundance

**Figure 2:**
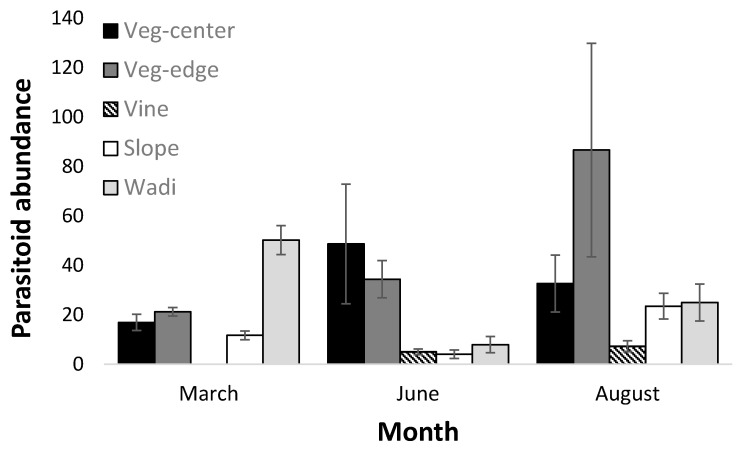
Total number of parasitoids (mean ± SE) in the different habitats and months. For simplicity, and due to the lack of significant difference, vine-center and vine-edge were pooled in this figure.

Parasitoid abundance was generally higher on the ground vegetation than on the grape foliage and differed between habitat types and months (Table 2, Figure 2). However, as is evident by the significant interaction between habitat and month (Table 2), the exact distribution of parasitoids depended on the timing in the season. Specifically, in March, parasitoids were most abundant in the wadi natural habitat; in June, they were more abundant in the vineyard ground vegetation habitats (both center and edge); and in August, they were most abundant at the vineyard edge (Figure 2). Plant species richness (see Table S2 for full species list) showed no effect on overall parasitoid abundance.

### 3.3. Dominant Species

**Figure 3:**
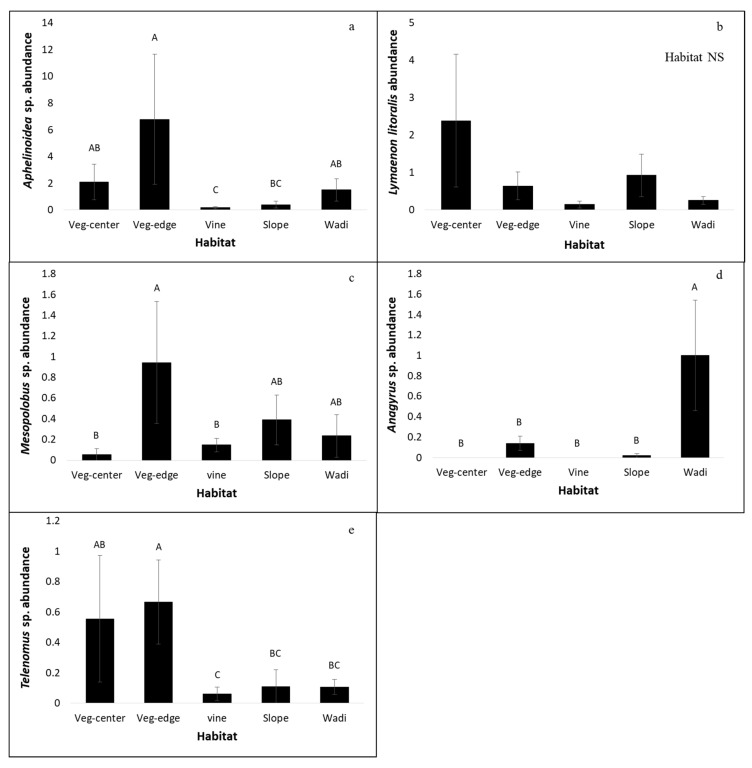
Total number of parasitoids (mean ± SE) of the five most dominant species in the different habitats. Letters represent statistical differences based on post-hoc tests. For simplicity, and due to lack of significant difference, vine-center and vine-edge were pooled in this figure.

Five dominant species accounted for 46% of the sampled parasitoids and were collected in sufficient numbers to enable us to analyze the environmental effects on their abundance (see statistical analyses). The abundance of four of these species was affected by season: *Aphelinoidea* sp., *Lymaenon litoralis* (previously *Gonatocerus litoralis*), and *Mesopolobus* sp. were collected mostly in June and August, while *Anagyrus* sp. was collected only in March (Table 3, not shown in the figure). Habitat type affected four of these species: *Aphelinoidea* sp. (accounting for 25% of the parasitoids collected) and *Mesopolobus* sp. were collected from all habitats, but were most abundant at ground vegetation of the vineyard edge; *Telenomus* sp., which was not affected by month, was found mainly in the ground vegetation of the agricultural habitats (center and edge); and *Anagyrus* sp. was found mainly in the wadi (Table 3, Figure 3). *Lymaenon litoralis*, which was not affected by habitat, was the only species that was positively associated with plant richness (Table 3).

### 3.4. Parasitoid Community Composition

Parasitoid community composition was significantly affected by the season (accounting for 7% of the variation) and the habitat (accounting for 4% of the variation) (Table 4). Specifically, post-hoc tests revealed significant differences in the comparisons of middle vs. slope (*p* = 0.049), edge vs. slope (*p* = 0.029), and center vs. wadi (*p* = 0.045), while the edge and wadi habitats did not significantly differ (*p* = 0.147). In addition, the composition was affected by plant species richness (2%) but did not significantly differ between the ground vegetation and the vines (Table 4).

## 4. Discussion

We studied parasitoid communities in six vineyards in a hyper-arid region in the Negev Desert. We predicted large differences in parasitoid abundance and composition between the vineyards and their surrounding natural habitats due to high environmental contrast. In accordance with our predictions, we found significant differences in parasitoid abundance and composition between the different habitats. However, habitat type explained only a small fraction of the variation in parasitoid community composition. This suggests that some parasitoid species exploited resources both within and outside of the vineyards. 

Parasitoid abundance generally increased throughout the growing season, as observed in previous studies previous studies, e.g., [47,48]. However, the distribution patterns of parasitoid abundances changed throughout the season—at the beginning of the season, higher abundances were found in the natural habitats, while later in the season, parasitoids were more abundant within the agricultural habitats. Similar seasonal patterns were previously demonstrated for spiders in desert wheat fields and were explained by the migration of spiders into the crop field throughout the season, combined with the high reproductive rates of some spider groups within the crop fields [9].

In the current study, strong seasonal rains that preceded the first sampling event contributed to the development of dense natural vegetation, especially on the wadi floor. These plants could have potentially attracted and supported parasitoids, providing them with floral nectar, insect hosts, and other resources [5,49,50,51,52]. Later in the season, herbaceous vegetation in the natural habitats had mostly dried out, and the vineyard habitat possibly became more attractive for parasitoids. This could be due to the irrigation inside the crop field, which despite weed control measures in several of the vineyards (see Table S1), maintained patches of green vegetation between the vine rows and along the crop edge. Indeed, non-crop natural vegetation was previously shown to support parasitoid abundance in vineyards in California [24], Australia [17,20], and northern Israel [53]. In particular, the edge habitat was shown to support a high abundance of parasitoids, including several of the dominant species, suggesting the potential importance of this habitat for biological control [2,24,54]. 

The parasitoid community included potential natural enemies of vineyard pests (e.g., moths, mealybugs, leafhoppers etc.; see Table 3), which are known to cause damage in this region (personal communications with vineyard farmers) and in vineyards in other areas in Israel (Shapira et al., 2018). Interestingly, the dominant parasitoid species varied in their spatial and temporal distribution patterns. For example, *Aphelinoidea* sp. (Trichogrammatidae) and *Lymaenon litoralis* (Mymaridae) (which together accounted for more than a third of the parasitoids sampled) were collected from both vineyard and natural habitats during the middle and end of the season. Species of these genera are known to attack leafhopper eggs and hence may be of importance for biological control, e.g., [55]. *Lymaenon litoralis* was the only dominant species affected by plant richness (and not by habitat type). Hence, this particular species could be potentially drawn to agricultural fields by means of plant diversification, e.g., [56]. Contrarily, *Anagyrus* sp. (Encyrtidae) was found mostly in the natural vegetation (wadi) and only in March. Although members of this genus are known to parasitize mealybugs, e.g., [57], the timing and location from which it was sampled suggest it is not likely to provide biological control services in this agroecosystem. It is difficult to determine whether the species of *Mesopolobus* and *Telenomus* that were abundant in our samples are natural enemies of vineyard pests, but species in both genera parasitize lepidopterans [33,58] and hence could potentially be beneficial.

Parasitoid species composition was significantly affected by the sampling month, as was demonstrated in previous studies [48,59]. In addition, parasitoid composition significantly differed between the natural and the crop habitats. However, while sampling month explained ~7% of the variation in parasitoid composition, habitat type only explained ~4% of this variation, and most of the dominant parasitoid species were found both within and outside of the vineyard (see Table 3). Moreover, the interaction between habitat and month had no effect on parasitoid species composition. These findings differed from a previous study in northern Israel, where the interaction between habitat and month explained a large proportion of the variation [60]; and from a study of South African vineyards, where remnant natural patches were shown to support a high abundance and diversity of parasitoids, but spillover into adjacent vineyards was limited [61]. 

The large overlap in parasitoid assemblages between the different habitats in our study may suggest that some aspects of the environmental contrast between crop and non-crop habitats were not as strong as initially assumed. For example, many plant species that occurred in the natural desert habitat were also present inside the vineyard (see Table S2), possibly attracting a similar assemblage of arthropods and their parasitoids, e.g., [52]. In addition, the sharp contrast in resource availability may sometimes not restrict, and perhaps may even promote arthropod movement between habitats. This may be due to the high relative abundance of arthropods spilling-over from the crops to the natural habitats following agriculture-related disturbances [62,63] and/or due to the high abundance of native arthropods seeking refuge in crop fields during the dry season [9]. The nearly nonexistent knowledge about parasitoids from this region, and from desert agroecosystems in general, makes it difficult to determine the source and movement patterns of the specific parasitoid species. Nevertheless, our data generally supports the notion that the surrounding natural habitats could potentially contribute to parasitoid recruitment and survival in this agroecosystem. 

Parasitoid composition did not differ between the non-crop ground vegetation and the grape foliage, suggesting that parasitoids attracted to the ground-cover vegetation could also visit the vines. Nevertheless, only a fraction of the parasitoids were sampled from the grape foliage. Similarly, Shapira et al. [60] found a similar composition but a lower abundance of parasitoids on grape foliage than on herbaceous vegetation in vineyards in northern Israel. This may suggest the limited importance of these parasitoids for the biological control of vine-pests. Alternatively, parasitoids may still attack potential vineyard pests, but spend less time in this habitat, which does not provide them with additional resources. 

## 5. Conclusions

In conclusion, despite the presumed high contrast in resource availability between the crop and non-crop habitats in the desert, our study suggests the potential importance of non-crop desert areas to the parasitoid community. Future investigations should consider yearly variations in parasitoid distribution patterns, the specific habitat and resource use of the different species, and their actual contribution to the biological control of the main pests, in this desert agroecosystem.

## Figures and Tables

**Table 1:** Number of samples taken at each location.

Location	Sampling Points	Timings	Vineyards	Total
Vineyard center, ground vegetation (Veg-center)	2	3 (March, June, August)	6	36
Vineyard center, grape foliage (Vine-center)	2	2 (June, August)	6	24
Vineyard edge, ground vegetation (Veg-edge)	2	3 (March, June, August)	6	36
Vineyard edge, grape foliage (Vine-edge)	2	2 (June, August)	6	24
Slope, natural vegetation (Slope)	2	3 (March, June, August)	6	36
Wadi, natural vegetation (Wadi)	2	3 (March, June, August)	6	36
Total				192

**Table 2:** Results of generalized linear mixed model (GLMM) testing of the effect of different environmental variables on parasitoid abundance. NS indicates a non-significant effect.

Tested Variable	χ^2^	df	*p*-Value
Month	10.41	2	<0.01
Habitat (Center/Edge/Slope/Wadi)	11.54	3	<0.001
Ground vegetation/Vine	30.36	1	<0.0001
Plant species richness	1.69	2	NS
Month × habitat	25.69	6	<0.0001

**Table 3:** Results of GLMM testing the effect of different environmental variables on the abundance of the most dominant parasitoid species. NA indicates that the sample size was too small to run the model.

Species (Family)	Potential Host Group (Primary Hosts)	N (% in Vineyards)	Results of GLMM		
			Month	Habitat	Plant Richness
*Aphelinoidea* sp. (Trichogrammatidae)	Leafhoppers [43]	399 (84%)	χ^2^_2_ = 24.29, *p* < 0.0001	χ^2^_4_ = 14.11, *p* < 0.01	χ^2^_1_ =1.62, NS
*Lymaenon litoralis* (Mymaridae)	Leafhoppers [44]	158 (73%)	χ^2^_2_ = 9.69, *p* < 0.01	χ^2^_4_ = 6.70, NS	χ^2^_1_ = 7.01, *p* < 0.01
*Mesopolobus* sp. (Pteromalidae)	Coleoptera, Diptera, Hymenoptera and Lepidoptera [33]	65 (66%)	χ^2^_2_ = 30.56, *p* < 0.0001	χ^2^_4_ = 13.44, *p* < 0.01	χ^2^_1_ =0.07, NS
*Telenomus* sp.1 (Platygastridae)	Lepidoptera and Heteroptera [25]	55 (85%)	χ^2^_2_ = 4.31, NS	χ^2^_4_ = 13.89, *p* < 0.01	χ^2^_1_ = 2.08, NS
*Anagyrus* sp. (Encyrtidae)	Pseudococcidae [33]	54 (9%)	χ^2^_2_ = 22.57, *p* < 0.0001	χ^2^_4_ = 18.75, *p* < 0.001	NA
*Paracentrobia* sp. (Trichogrammatidae)	Auchenorrhyncha [34]	36 (100%)	NA		
*Eurytoma* (Eurytomidae)	Coleoptera, Diptera, Hymenoptera and Lepidoptera [33]	35 (54%)	NA		
*Diglyphus isaea* (Eulophidae)	Agromyzidae [45]	34 (35%)	NA		
*Pseudoligosita* sp. (Trichogrammatidae)	Auchenorrhyncha [34]	33 (94%)	NA		
*Telenomus* sp.2 (Platygastridae)	Lepidoptera and Heteroptera [25]	33 (94%)	NA		
*Telenomus* sp.3 (Platygastridae)	Lepidoptera and Heteroptera [25]	33 (97%)	NA		
*Tumidiclava tamariska* (Trichogrammatidae)	Cicadellidae [46]	30 (83%)	NA		

**Table 4:** Results of PERMANOVA testing of the effect of different environmental variables on parasitoid species composition.

Tested Variable	df	F	R^2^	*p*-Value
Month	2, 69	3.083	0.069	0.0001
Habitat (Center/Edge/Slope/Wadi)	3, 69	1.238	0.042	0.024
Vine/Veg	1, 69	1.303	0.001	NS
Plant species richness	1, 72	2.015	0.023	<0.001
Month × Habitat	6, 69	1.049	0.007	NS

## Supplementary Materials

The following are available online at https://www.mdpi.com/2075-4450/11/9/580/s1, Table S1. Location and characteristics of the six sampled vineyards, Table S2: Plant species occurring at the different crop-related and adjacent natural habitats.
