# Supplementary material for: Parasitoid Abundance and Community Composition in Desert Vineyards and Their Adjacent Natural Habitats
Source: Insects. 2020 Sep 1;11(9):580. doi: 10.3390/insects11090580 (PMC7565741; doi:10.3390/insects11090580)
Supplement: Supplementary file 1 [file insects-11-00580-s001.pdf]

Table S1. Location and characteristics of the six sampled vineyards.

Location Coordinates	Area (Dunam)	Age (Oldest Vines)	Varieties	Pesticides	Herbicides	Other Chemicals	Main Pests
30.61347, 34.74881	12	5	Cabernet Sauvignon, Malbec, Merlot, Chenin Blanc, Suvignon Blanc, Granach	No	No	No	No major pests
30.61639, 34.75466	84	9	Cabernet Sauvignon, Chardonnay, Syrah, Granach, Petit Syrah, chenin Blanc,	No	Only under the trunk	Fungicides	*Lobesia botrana*, aphids
30.62567, 34.76206	48	4	Chardonnay, Sira, Muskat, Cabarnet Sauvignon, Malbek, Cabernet Franc	Only during outbreaks	Only during outbreaks	No	No major pests
30.64726, 34.78892	450	6	Cabarnet Sauvignion, Merlot, Chardonnay, Petit Verdo	Yes	Yes	Fungicides	*Lobesia botrana*, mealybugs, leafhoppers
30.64008, 34.79607	12	9	Cabarnet, Petit	No	Yes	No	No major pests
30.67201, 34.80899	15	10	Cabarnet, Merlot, Petit Zigfendral,Chardonnay	No	No	No	leafhoppers, aphids

Table S2. Plant species occurring at the different crop-related and adjacent natural habitats.

Family	Plant Species	Number of Plots with Plant Occurrence			
		Border	Middle	Slope	Wadi
Aizoaceae	*Mesembryanthemum nodiflorum*	1			
Amaranthaceae	*Amaranthus sp.*	4	4		
Boraginaceae	*Echium sp.*				1
Caryophyllaceae	*Gymnocarpos decander*		1	3	
Caryophyllaceae	*Gypsophila arabica*				1
Chenopodiaceae	*Agathophora alopecuroides*			5	
Chenopodiaceae	*Anabasis articulata*			6	2
Chenopodiaceae	*Anabasis syriaca*	10	8	2	12
Chenopodiaceae	*Atriplex sp.*	14	13		1
Chenopodiaceae	*Atriplex sp.2*		2		
Chenopodiaceae	*Bassia arabica*	2		1	
Chenopodiaceae	*Bassia indica*	9	7		
Chenopodiaceae	*Chenopodium sp.*	2			
Chenopodiaceae	*Halothamnus lancifolius*			2	1
Chenopodiaceae	*Noaea mucronata*			12	1
Chenopodiaceae	*Salsola incanescens*	4			3
Chenopodiaceae	*Salsola inermis*	8	6	7	2
Chenopodiaceae	*Salsola sp.*	2			1
Chenopodiaceae	*Salsola tragus*	3	1	1	
Chenopodiaceae	*Salsola vermiculata*	1		1	
Compositae	*Achillea fragrantissima*				4
Compositae	*Anthemis sp.*	1			1
Compositae	*Artemisia sieberi*		1	7	16
Compositae	*Asteraceae sp.*		2		1
Compositae	*Centaurea sp.*		1		1
Compositae	*Erigeron sp.*	4	7		
Compositae	*Pulicaria undulata*	1	1		
Compositae	*Sonchus oleraceus*	4	3		
Convolvulaceae	*Convolvulus althaeoides*	1	4		1
Cruciferae (Brassicaceae)	*Erucaria microcarpa*	1			
Cruciferae (Brassicaceae)	*Matthiola livida*		1		
Cruciferae (Brassicaceae)	*Moricandia nitens*	2	1		
Euphorbiaceae	*Andrachne telephioides*		3		
Euphorbiaceae	*Chrozophora tinctoria*		3		
Gramineae (Poaceae)	*Cynodon dactylon*	2	6		
Gramineae (Poaceae)	*Gastridium sp1.*	*2*		*3*	*3*
Gramineae (Poaceae)	*Gastridium sp2.*		1		1
Gramineae (Poaceae)	*Gastridium sp3.*				1
Gramineae (Poaceae)	*Gastridium sp4.*	2	3		
Labiatae (Lamiaceae)	*Ballota undulata*		1		1
Labiatae (Lamiaceae)	*Marrubium alyssum*	4			
Labiatae (Lamiaceae)	*Stachys aegyptiaca*			1	

Malvaceae	*Malva sp.*	5	2		
Papilionaceae	*Medicago radiata*		1		
Plantaginaceae	*Plantago sp.*				4
Plumbaginaceae	*Limonium pruinosum*			2	
Polygonaceae	*Polygonum equisetiforme*	1	1		
Resedaceae	*Reseda sp.*	1	1		
Scrophulariaceae	*Scrophularia sp.*				1
Scrophulariaceae	*Verbascum sinaiticum*				1
Scrophulariaceae	*Verbascum sp.*		1		2
Solanaceae	*Solanum nigrum*	1	1		
Solanaceae	*Withania somnifera*	1			
Tamaricaceae	*Reaumuria hirtella*	2		12	3
Tamaricaceae	*Reaumuria negevensis*			1	
Thymelaeaceae	*Thymelaea hirsuta*		1	1	2
Zygophyllaceae	*Peganum harmala*	2			11
Zygophyllaceae	*Zygophyllum dumosum*			4	
